# Light-Responsive PLGA Microparticles for On-Demand Vancomycin Release and Enhanced Antibacterial Efficiency

**DOI:** 10.3390/pharmaceutics17081007

**Published:** 2025-08-01

**Authors:** Mishal Pokharel, Abid Neron, Amit Kumar Dey, Aishwarya Raksha Siddharthan, Menaka Konara, Md Mainuddin Sagar, Tracie Ferreira, Kihan Park

**Affiliations:** 1 Department of Biongineering, University of Massachusetts Dartmouth, Dartmouth, MA 02740, USA; aneron@umassd.edu (A.N.);; 2 Department of Mechanical Engineering, University of Massachusetts Dartmouth, Dartmouth, MA 02740, USA

**Keywords:** near-infrared light, microparticles, antibiotic, drug delivery, controlled release, chitosan coating

## Abstract

**Background:** A precise drug delivery system enables the optimization of treatments with minimal side effects if it can deliver medication only when activated by a specific light source. This study presents a controlled drug delivery system based on poly(lactic-co-glycolic acid) (PLGA) microparticles (MPs) designed for the sustained release of vancomycin hydrochloride. **Methods:** The MPs were co-loaded with indocyanine green (ICG), a near-infrared (NIR) responsive agent, and fabricated via the double emulsion method.They were characterized for stability, surface modification, biocompatibility, and antibacterial efficacy. **Results:** Dynamic light scattering and zeta potential analyses confirmed significant increases in particle size and surface charge reversal following chitosan coating. Scanning electron microscopy revealed uniform morphology in uncoated MPs (1–10 μm) and irregular surfaces post-coating. Stability tests demonstrated drug retention for up to 180 days. Among formulations, PVI1 exhibited the highest yield (76.67 ± 1.3%) and encapsulation efficiency (56.2 ± 1.95%). NIR irradiation (808 nm) enhanced drug release kinetics, with formulation PVI4 achieving over 48.9% release, resulting in improved antibacterial activity. Chitosan-coated MPs (e.g., PVI4-C) effectively suppressed drug release without NIR light for up to 8 h, with cumulative release reaching only 10.89%. Without NIR light, bacterial colonies exceeded 1000 CFU; NIR-triggered release reduced them below 120 CFU. Drug release data fitted best with the zero-order and Korsmeyer–Peppas models, suggesting a combination of diffusion-controlled and constant-rate release behavior. **Conclusions:** These results demonstrate the promise of chitosan-coated NIR-responsive PLGA MPs for precise, on-demand antibiotic delivery and improved antibacterial performance.

## 1. Introduction

Microparticles represent a significant development in biomedical engineering, providing promising applications in targeted drug delivery, tissue regeneration, and wound healing. Studies have demonstrated that microparticles can be designed with precise characteristics to improve the effectiveness of drug delivery [1,2]. The size of the microparticles runs from 1 to 1000 μm and is used in drug delivery methods where multiple particles are used to encapsulate large molecules. This is done to treat different medical ailments such as ocular disorders, cancer, cardiac conditions, and inflammation [3].

Poly (lactic-co-glycolic acid) (PLGA) is a commonly used material in the realm of biomedical engineering. It is often used to encapsulate and deliver therapeutic drugs, proteins, and antigens over an extended duration [4]. PLGA is known for its exceptional biodegradability and its compatibility with biological tissues. It has been deemed safe by the United States Food and Drug Administration (FDA) and the European Medicines Agency (EMA) [5]. PLGA microparticles enable controlled drug release by diffusion and erosion mechanisms and can be tailored for time-controlled drug delivery [6]. PLGA is considered to be an efficient polymer for drug delivery [7,8,9].

Efficient release of drugs using microparticles is achieved with the use of different types of trigger techniques. These triggers range from ultrasound [10], light [11], electric [12], and magnetic fields [13] to radio frequency [14].

Drug pharmacodynamics also plays a crucial role in achieving the desired therapeutic outcome. The optimization of drug release should be kept under consideration for efficient drug delivery [15]. The controlled release of drugs has immense importance, besides the target specificity. Controlled drug release from microparticles is a promising approach in pharmaceutical technology. It aims to improve the effectiveness of drugs while reducing any negative effects they may have. When drugs are placed inside micro/nanoparticles made from materials that can break down naturally and are safe for the body, like PLGA, the drugs can be released slowly over some time through spreading out, breaking down, and wearing away processes [16,17,18]. This approach helps to maintain a consistent therapeutic effect, reduces the number of times medication needs to be taken, and makes it easier for patients to follow their treatment plan. There are many different ways that this technology can be used in the medical field. Some examples include using it for cancer treatment [19], managing chronic diseases [20], and developing vaccines [21]. Studies are currently being conducted widely on improving the way drugs are delivered to the body. New developments with nanomaterials [22,23,24] and nanotechnology [25,26] are expected to create more advanced systems that have the capability to release drugs with a great precision.

Near-infrared (NIR) light is being increasingly recognized as a valuable tool for regulating the release of drugs in different drug delivery systems. Research has demonstrated that NIR light can activate the release of drugs by using photothermal effects. This allows for accurate and timely delivery of drugs as needed [27,28,29]. Extensive research is being carried out to develop innovative and advanced drug delivery systems that respond to NIR light stimuli. Zhang et al. explored actively loaded doxorubicin and gold nanorod liposomes that respond to NIR, as it triggers photothermal conversion [30]. Likewise, Salazar Sandoval et al. synthesized nanocarriers with gold nanorods for NIR-triggered drug release [31]. This method provides a sophisticated and controlled way to deliver drugs via the PLGA microparticles, which is especially useful in cancer treatment studies [32,33]. This technology involves incorporating photothermal agents like gold nanoparticles or carbon nanotubes into the PLGA matrix. When these agents are exposed to NIR light, they convert the light into heat in a specific area, causing the PLGA to break down. This results in the release of drugs in a controlled manner [34,35,36].

The benefits of this NIR-activated drug release are that it allows for drug release without invasive procedures, enables NIR light to penetrate deep into tissues, and offers the ability to target specific locations within the body [37,38,39]. This is especially helpful in applications like cancer treatment where targeted chemotherapy can be conducted on tumor sites [40,41,42]. Similarly, the release of antibiotics can be precisely controlled to maintain a high concentration of the drug at the site of infection for infection control purposes. These microparticles can then be released in response to NIR light, which enables precise control over when and where delivery occurs [43,44]. However, this method has challenges of its own. They involve ensuring that the photothermal agents are safe for use in the body and remain stable, controlling the heat to avoid harming the tissues, and increasing production while still meeting quality and regulatory standards. This has been shown in several studies on NIR-triggered PLGA systems in cancer therapy [43,45] and bone regeneration [46].

Vancomycin, a tricyclic glycopeptide antibiotic, though not used as a direct therapeutic for melanoma, plays an important role in combating infections in patients who undergo surgery or immunotherapy [47]. Its ability to combat resistant bacterial strains helps prevent complications during cancer treatment [48]. A targeted approach, like the use of NIR-responsive PLGA microparticles for the drug release directly at the infection sites, can enhance antibacterial effectiveness while minimizing side effects. Indocyanine Green (ICG), a NIR-responsive dye, serves as a critical component in this context. ICG’s unique ability to respond to NIR light allows it to trigger precise, controlled drug release [49]. Upon exposure to NIR light, ICG generates heat, activating the release of encapsulated drugs like vancomycin hydrochloride (Van.HCl), offering a more targeted therapeutic approach. ICG-loaded microparticles maintain a constant drug release rate, ensuring steady therapeutic levels over time [50].

In recent years, several studies have explored the use of NIR light for developing efficient drug delivery systems [51,52,53,54,55,56,57]. While these studies demonstrate diverse approaches to light responsiveness, some challenges remain, particularly in achieving precise controllability in drug delivery. The originality of this research stands in the use of NIR-responsive PLGA microparticles to deliver the antibiotic vancomycin with optimum control. A significant development in this study is the utilization of ICG in conjunction with a chitosan coating, which allows the microparticles to respond to NIR light without excessively releasing the content. This approach enables precise, light-controlled release of the antibiotic, providing a non-invasive method to deliver medication directly to infection sites, reducing the need for repeated doses. This study thus carves a path for a more targeted and controlled drug delivery, offering a promising tool in the niche of drug delivery and therapeutics.

## 2. Materials and Methodology

### 2.1. Materials

Poly (lactic-co-glycolic acid) acid endcap (60:40 LA:GA, Mn: 15,000–25,000 Da) was purchased from Akina Inc. (PolySci Tech), West Lafayette, IN, USA. Please check in the whole manuscript and ensure the necessary information have been provided. Poly(vinyl alcohol) (PVA) (87–90% hydrolyzed with an avg. molecular weight of 30,000–70,000), dichloromethane (DCM), Low Molecular Weight Chitosan (50–190 kDa, 75–85% deactylated), Endotoxin-Free Ultra Pure Water, and Vancomycin Hydrochloride (Certified Reference Material was produced and certified per [58,59] were obtained from Millipore Sigma, Burlington, MA, USA. Indocyanine green was purchased from Adooq Biosciences, Irvine, CA, USA. All acquired chemicals are of analytical grade and were used without any further purification.

### 2.2. Cell Lines and Cell Culture

The A375 cells (CRL-1619™) were purchased from the American Type Culture Collection (ATCC). They were isolated from the skin of a 54-year-old female patient with malignant melanoma. The cells were cultured in Dulbecco’s modified eagle medium (DMEM; Millipore Sigma, Burlington, MA, USA) supplemented with 10% fetal bovine serum (FBS; Gibco, Invitrogen, Waltham, MA, USA), 1% penicillin-streptomycin (Gibco, Invitrogen, Waltham, MA, USA), and maintained at 37 °C in a humidified atmosphere containing 5% CO_2_.

### 2.3. Methodology

#### 2.3.1. Fabrication of PLGA-ICG-Van.HCl Microparticles

**Figure 1:**
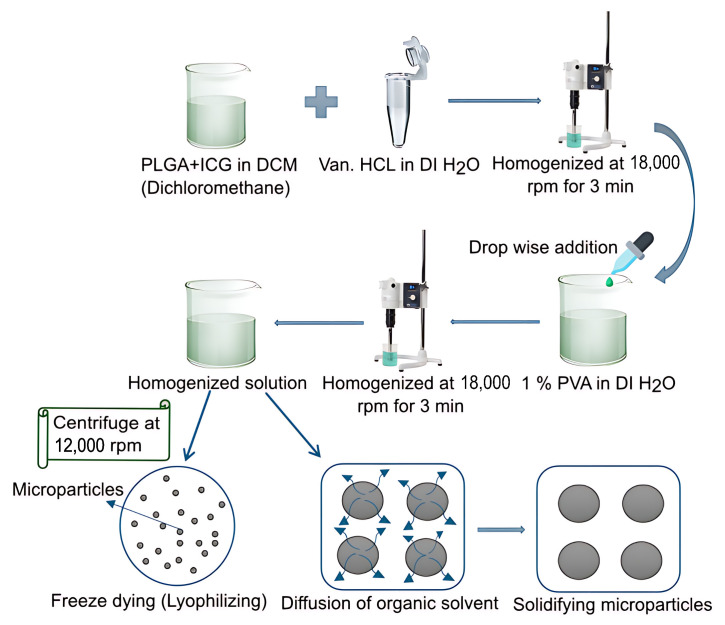
A schematic of the fabrication procedure for NIR-responsive PLGA microparticles.

The PLGA-ICG-Van.HCl particles were fabricated using the double emulsion method [60] with slight modifications as shown in Figure 1.

A predetermined amount of PLGA and ICG was dissolved in DCM making the oil phase (O). Similarly, Van.HCl was dissolved in distilled water, making the first water phase (W_1_). The two solutions were mixed by homogenizing at 18,000 rpm for 3 min. The homogenized solution was then added to 60 mL of 1% PVA and then homogenized at 18,000 rpm for 3 min. The final solution was then left stirring at 700 rpm and 25 °C. To facilitate particle solidification, the solvent was allowed to evaporate for 4 h. Subsequently, the microparticles were centrifuged at 12,000 rpm for 20 min at 4 °C, washed twice with distilled water, and lyophilized for 24 h at −40 °C and 0.04 mbar. The lyophilized particles were stored in air-tight containers in a −20 °C freezer. Various formulations were developed with different drugs, polymers, and ICG content while maintaining a constant water phase, as shown in Table 1. The rationale behind using different formulations was to investigate the impact of varying drug, polymer, and ICG concentrations on the desired properties of the final product.

The initial three formulations, namely PVI1, PVI2, and PVI3, involved keeping the ICG content constant while altering the drug and polymer content. This approach allowed for evaluating the influence of different drug concentrations on the characteristics of the formulation, such as drug release rate, stability, and overall performance. In the subsequent three formulations, PVI4, PVI5, and PVI6, the focus shifted to keeping the polymer and drug content constant while changing the ICG content. By doing so, the specific impact of varying ICG concentrations on critical aspects, such as particle size, encapsulation efficiency, drug–polymer interactions, and temperature changes when exposed to NIR light could be assessed. P7 and PI were fabricated using solely PLGA and PLGA with ICG, respectively, to serve as a control for all experiments to account for PLGA and ICG in the observed microparticle behavior.

#### 2.3.2. Coating of Microparticles with Chitosan

The fabricated microparticles were coated using a modified NHS-EDC method [61], incorporating slight adjustments to minimize cargo loss and enhance the stability of the functionalized surface. To prepare a 1% (*w*/*v*) chitosan solution, 0.5 g of chitosan was dissolved in 50 mL of a 1% (*v*/*v*) acetic acid solution. For the covalent conjugation of chitosan to PLGA, the polymer was first precipitated by adjusting the pH to ∼7.4 using 1.0 M sodium hydroxide. The precipitate was then homogenized at 24,000 rpm for 1 min, preparing it for chemical conjugation.

To coat the chitosan onto the microparticles, 100 mg of microparticles were resuspended in 5 mL of 100 mM MES buffer (pH 5.5) and sonicated in an ultrasonic bath for 30 s. The carboxyl groups on the PLGA particles were activated by preparing a 0.1 M solution of N-hydroxy succinimide (NHS) and 1-(3-dimethyl aminopropyl)-3-ethyl carbodiimide hydrochloride (EDC) by dissolving 115 mg of NHS and 190 mg of EDC in 10 mL of HEPES buffer (20 mM, pH 7.4). To the microparticle suspension in MES buffer, 2 mL of this NHS/EDC solution was added, followed by stirring at 500 rpm for 3 h at room temperature. The suspension was then centrifuged at 12,000 rpm for 15 min at 4 °C. If ICG was present, the reaction was protected from light by covering it with aluminum foil. After centrifugation, the supernatant was discarded, and the microparticles were washed with 5 mL of distilled water, then resuspended in 5 mL of HEPES/NaOH buffer and sonicated for 30 s. Then, 3 mL of chitosan suspension was added to the microparticle suspension, and the mixture was stirred at 1500 rpm for 8 h at room temperature. Finally, the chitosan-conjugated microparticles were recovered by centrifugation at 12,000 rpm for 20 min at 4 °C, washed twice with distilled water, lyophilized, and stored at 4 °C until use.

The fabricated microparticles, PVI4, PVI5, and PVI6, as presented in Table 1, were selected for chitosan coating due to their uniform drug and polymer composition. The chitosan-coated particles were designated as PVI4-C, PVI5-C, and PVI6-C, where “C” signifies the presence of the chitosan coating. PVI2 and PI were coated with chitosan for comparison purposes and have been labeled as PVI2-C and PI-C, respectively.

#### 2.3.3. Dynamic Light Scattering (DLS) and Zeta Potential Analysis

The hydrodynamic diameter and polydispersity index (PDI) of PLGA-Van.HCl-ICG microparticles, both unmodified and surface-conjugated (denoted as PVI and PVI-C, respectively), were determined using Dynamic Light Scattering (DLS) on a Malvern Zetasizer Advance Ultra (Malvern Panalytical Ltd., Malvern, UK). Measurements were performed at 25 °C using backscatter detection at a 173° angle. Samples were diluted in deionized water to an appropriate concentration to avoid multiple scattering effects. Zeta potential was measured on the same instrument using electrophoretic light scattering with a folded capillary cell (DTS1070). The same microparticle suspensions were diluted and equilibrated at room temperature.

#### 2.3.4. Production Yield (PY) of the Microparticles

Production yield is an important parameter to judge the efficiency of the method used for the preparation of microparticles. It is the percentage of the total mass of the product obtained from the total mass used, which can be calculated from the following formula:

(1) $$PY=\frac{\mathrm{Practical}\mathrm{Mass}\mathrm{of}\mathrm{Microparticles}}{\mathrm{Theoretical}\mathrm{Mass}(\mathrm{Drug}+\mathrm{Polymer})}*100$$

#### 2.3.5. Percent Drug Content and Entrapment Efficiency

The quantification of percent drug content and encapsulation efficiency for the prepared microparticles involved weighing samples of the microparticles and dissolving them in 1 M NaOH. The solution was stirred at 300 rpm for 24 h and then filtered using a 0.22 μm syringe filter. The coated microparticles were subjected to sonication at 30 °C for 30 min before stirring for 24 h. The resulting solution was analyzed at a wavelength of 280 nm. The percent drug content and entrapment efficiency were determined using the following equations:

(2) $$\mathrm{Drug}\;\mathrm{Content}\left(\%\right)=\frac{M_{i}}{M_{\mathrm{mp}}}\times 100$$

(3) $$\mathrm{Entrapment}\;\mathrm{Efficiency}\left(\%\right)=\frac{M_{i}}{M_{t}}\times 100$$

where M*_i_* is the amount of drug present in the fabricated microparticles, $M_{mp}$ is the weight of the fabricated microparticles, and M*_t_* is the theoretical amount of drug in the microparticles.

#### 2.3.6. Characterization of Fabricated Microparticles

An absorbance test was done to confirm the difference in ICG for the formulations (PVI1, PVI2, PVI3, PVI4, PVI5, PVI6) in the fabricated microparticles. This was done by dispersing 5 mg of microparticles in 1M NaOH. The solution was stirred at 300 rpm for 24 h and then filtered using a 0.22 μm syringe filter. The resulting solution was analyzed at a wavelength range of 300–900 nm. Furthermore, PVI4, PVI5, and, PVI6 microparticles dispersed in distilled water, along with control samples (phosphate-buffered saline (PBS), P7, and 5 mM ICG) were irradiated with NIR light (808 nm at 0.3 W/cm^2^), and their respective temperature changes were recorded over time using a thermal probe. To analyze the shape and size of the fabricated microparticles a HITACHI 3700n-VP Scanning Electron Microscopy (SEM) was used for microparticle observation. A low voltage of 3 kV and a magnification of ×3.5 k and ×6 k were employed to capture the images. The microparticle samples were prepared by dispersing lyophilized powders onto carbon tape mounted on a 26 mm SEM stub. The samples were then coated with gold using a Denton Vacuum Desk IV (Denton Vacuum LLC, Moorestown, NJ, USA) for 45 s at 30% power, resulting in a conductive layer approximately 5.56 nm thick. Differential Scanning Calorimetry (DSC) analysis was done using the Thermal Analyzer DC25. For the uncoated microparticles, the initial temperature was kept at 0 °C after which the temperature was ramped up at 10 °C per minute to a final temperature of 350 °C. The final temperature was increased to 400 °C for the coated microparticles. The data were collected using the TRIOS software v5.7.2.101, transported to Microsoft Excel, and plotted using OriginPro2024b. Long-term stability is a crucial factor in evaluating the efficacy of drug delivery systems. To assess stability over time, the percentage of drug content (DC) and encapsulation efficiency (EE) were measured 180 days post-fabrication. Additionally, to ensure that the microparticle structure and morphology remained unaffected, SEM imaging was performed at both 90 and 180 days after fabrication. These analyses help determine potential degradation, structural integrity, and sustained drug retention over extended periods.

#### 2.3.7. Bond Detection by Fourier-Transform Infrared Spectroscopy (FTIR)

An infrared spectrometer (Cary 630 FTIR, Agilent, Santa Clara, CA, USA) was employed to analyze the samples’ infrared spectrum. The sample was positioned onto the instrument, and the Attenuated Total Reflectance (ATR) objective was brought into contact with the sample surface. Subsequently, absorbance readings were recorded from the sample surface. The spectra were recorded from 500 to 4000 cm^−1^, and the obtained numerical values were transferred to Microsoft Excel and plotted using OriginPro 2024b for graphical representation. The IR fingerprint region of the amide bond was obtained from the literature and was compared with the IR spectrum of chitosan-conjugated particles.

#### 2.3.8. Wettability Test

To evaluate the wettability of our PLGA-based materials, we conducted a contact-angle measurement study using thin films as a model system. Since direct measurement of the contact angle on individual microparticles is challenging due to their small size and irregular morphology, thin films were used to approximate the surface wettability of the bulk material. For film preparation, PLGA was dissolved in DCM to create a 10 wt.% solution. Similarly, to make a PLGA-ICG film, ICG was dissolved in the same PLGA solution to achieve a final concentration of 1 mM ICG. The solutions were mixed thoroughly to ensure homogeneity before film formation. Then, 100 μL of the solution was carefully pipetted onto a cleaned glass slide, ensuring even distribution across the surface. The solvent was allowed to evaporate for 4 h under ambient conditions, after which the films were vacuum-dried at room temperature for a couple of hours to remove any residual solvent. The obtained PLGA and PLGA-ICG films had a uniform, glossy appearance, indicating successful film formation. Once dried, the glass slides containing the films were transferred to a 6-well plate, with each well containing 2 mL of fresh, sterile phosphate-buffered saline (PBS) (pH 7.4). A batch of PLGA and PLGA-ICG films was then exposed to NIR light (808 nm) at 0.3 W/cm^2^ for 10 min to evaluate the potential photothermal effects on surface wettability. Following irradiation, the 6-well plates were sealed with parafilm and placed inside an incubator at 37 °C with controlled humidity to simulate in vitro conditions. Contact-angle readings were taken at pre-determined time intervals using a Goniometer (rame-hart instruments) to monitor changes in surface hydrophilicity over time. The use of films allowed us to model the wettability behavior of the microparticles in biological systems, providing insight into how their surface properties might evolve upon exposure to physiological conditions or external stimuli.

#### 2.3.9. Drug Release Assay

To test for drug release from the microparticles, 10 mg of fabricated PLGA microparticles containing Van.HCl and NIR-responsive materials were placed into a 2 mL solution of PBS with a pH of 7.4 inside a Cytivia dialysis kit with a cut-off of 8 kDa. The Cytivia kit containing the solution was vortexed for dispersion of the microparticles and placed in a beaker containing 18 mL of the same PBS at 37 °C and stirred at 100 rpm. After an hour, the microparticles were exposed to NIR laser at 0.3 W/cm^2^ and 808 nm for 30 min. Then, 1 mL of the sample was taken out at specific time intervals. To maintain the sink condition, 1 mL of PBS was added to the solution. The readings were taken using a ThermoFisher Genysis 50 UV-Visible Spectrophotometer at 280 nm. For the drug release assay and the subsequent antibacterial tests (Section 2.3.10), the irradiated microparticles are designated with an “N” at the end of their formulation name (e.g., PVI4 N, PVI5 N, PVI4-C N, etc.). The concentration of Van.HCl was obtained from a calibration curve that was obtained before starting the drug release.

#### 2.3.10. Antibacterial Tests

Escherichia coli S17-1 (*E. coli*) was cultured in lysogeny broth (LB) (Sigma Life Science) and incubated overnight at 37 °C at 250 rpm for 24 h. Fresh bacterial cultures were prepared for each antibacterial test using the previously refreshed culture. For the antibacterial test, a new batch of bacteria was cultured overnight until reaching an optical density at 600 nm (OD600) of 0.5, corresponding to approximately 8 × 10^8^ cells/mL. In a microcentrifuge tube, 10 mg of microparticles were combined with 1 mL of LB and inoculated with 10 μL of the bacterial culture. Each microparticle group was tested in duplicate: one sample was exposed to NIR light at 0.3 W/cm^2^ power for 30 min, while the other sample was not exposed to NIR light. The unexposed sample was kept at room temperature for 30 min to ensure consistency. Both samples were then incubated at 37 °C for 2 h. After incubation, 100 μL of each sample was spread onto an LB agar plate (Fisher BioReagents, Fisher Sci., Waltham, MA, USA) and incubated at 37 °C for 24 h to allow bacterial growth for visualization. A control group, prepared using the same protocol without the addition of any microparticles, was included for comparative analysis.

#### 2.3.11. alamarBlue Biocompatibility Assay

Each well of a 96-well plate was seeded with 6000 cells and allowed to attach for 36 h. After this period, the media was discarded, and the cells were washed with PBS. The cells were then treated with 0.2 mL of lyophilized microparticles (PVI4, PVI4-C, PVI5, PVI5-C), each at a concentration of 500 μg/mL, dispersed in DMEM containing 1% penicillin-streptomycin and 10% FBS. Half of the wells containing microparticles and cells were exposed to NIR light at 0.3 W/cm^2^ power and an 808 nm wavelength, while the other half remained unexposed. A 3% (*v*/*v*) in cell culture media) dimethyl sulfoxide (DMSO) solution was used as a positive control, and untreated cells served as the negative control. After 24 h, the microparticles were removed and the alamarBlue™ working solution was added to the wells. The cells were incubated for 2 h, and then transferred to a black 96-well plate for fluorescence measurements at excitation and emission wavelengths of 560 nm and 590 nm, respectively.

#### 2.3.12. Zebrafish Embryo Toxicity (ZET) Model

To assess the biocompatibility of the fabricated microparticles, zebrafish embryos were collected 4-5 h post fertilization (hpf). For each study, 15 healthy embryos were transferred into a 2 mL Eppendorf tube. A solution of freeze-dried microparticles (PVI4) of 250 μg/mL was prepared. The ethanol (2% (*v*/*v*)) and caffeine (Caff) (200 ppm) served as a positive control (PC), and untreated zebrafish in regular fish water with methylene blue served as a negative control (NC). The group exposed to ethanol is designated as PC, while PCN represents the same group subjected to NIR light stimulation. Similarly, Caff and Caff N denote the caffeine-exposed groups without and with NIR light stimulation, respectively, and NC and NCN represent the control group under the same conditions. Then, 1 mL of each solution was transferred into Eppendorf tubes. All groups had one group exposed to NIR light (808 nm, 0.3 W/cm^2^ for 30 min), while the other was unexposed. After exposure, all embryos were transferred to a 6-well plate containing 4 mL of the same media (fish water for the negative control, 2% (*v*/*v*) ethanol for the positive control, 250 μg/mL of freeze-dried PVI4 microparticles, 200 ppm caffeine solution). The hatching and survival rate of the zebrafish embryos were evaluated by direct microscopic observation.

#### 2.3.13. Statistical Analysis

Unless otherwise specified, data are expressed as mean ± standard deviation (mean ± SD). Statistical analyses were conducted using GraphPad Prism 8, with significance determined by analysis of variance (ANOVA). A *p*-value of less than 0.01 was considered statistically significant. Unless otherwise stated, all the above experiments were performed in triplicate (n = 3), except for the absorbance readings.

## 3. Results and Discussion

### 3.1. Dynamic Light Scattering (DLS) and Zeta Potential Analysis

**Figure 2:**
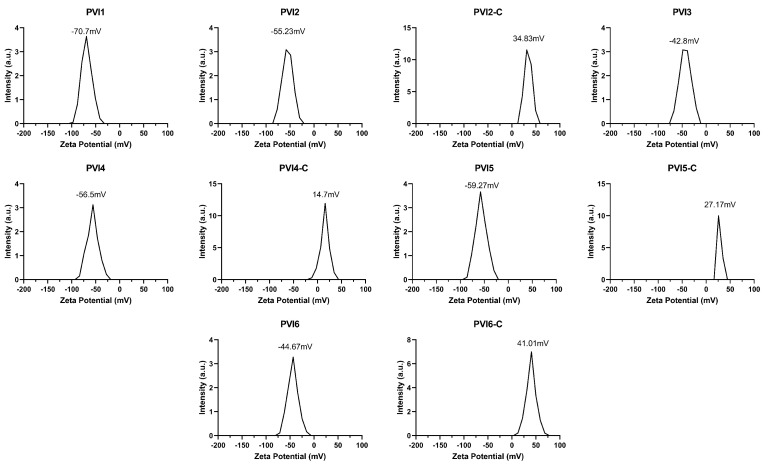
Zeta potential readings of various formulations of fabricated PLGA microparticles, including both uncoated and chitosan-coated samples. The letter “C” in the formulation names denotes chitosan coating (e.g., PVI4-C represents the PVI4 formulation coated with chitosan).

Zeta potential analysis shown in Figure 2 and Table 2 revealed strong negative charges for unmodified PLGA-Van.HCl-ICG microparticles (−44 to −70.7 mV), suggesting excellent colloidal stability. Post-conjugation particles (PV-C series) showed significantly reduced or inverted zeta potentials (up to +40 mV), confirming successful surface modification. DLS analysis shown in Table 2 revealed that the Z-average diameter of the microparticle formulations ranged from 2.26 μm (PVI1) to 7.75 μm (PVI4-C), with particle sizes generally increasing upon chitosan coating. Uncoated formulations such as PVI1, PVI2, and PVI4 exhibited smaller sizes (approximately 2.3–2.4 µm), while PVI3, PVI5, and PVI6 showed larger diameters (3.3–6.8 µm), indicating variation in polymer composition or aggregation tendency. Chitosan-coated formulations (denoted “-C”) consistently displayed substantial increases in particle size compared to their uncoated counterparts, for example, PVI2-C (5.71 μm) vs. PVI2 (2.44 μm), PVI4-C (7.75 μm) vs. PVI4 (2.34 μm), and PVI5-C (7.7 μm) vs. PVI5 (6.78 μm), suggesting successful surface coating and potential interparticle bridging. Polydispersity Index (PI) values ranged from 0.196 (PVI4) to 0.755 (PVI4-C), with uncoated particles such as PVI1 (0.284), PVI3 (0.264), PVI5 (0.324), and PVI6 (0.293) generally exhibiting moderate polydispersity, indicative of relatively uniform particle populations. In contrast, chitosan-coated variants like PVI4-C (0.755), PVI5-C (0.686), and PVI2-C (0.454) showed higher PIs, pointing to broader particle size distributions likely due to variable coating thickness or aggregation during the coating process [62]. These findings are consistent with literature reports that chitosan coating increases particle size and heterogeneity due to the formation of a hydrated polymer shell and potential electrostatic interactions [63]. Moreover, the overall size range exceeds the ideal range for DLS accuracy but remains useful for comparative analysis; the acceptable PI values (all <1.0) also support the reliability of the measurements [64]. Together, the data indicate that chitosan coating effectively modifies the physical characteristics of the microparticles, which may influence their biological performance in drug delivery applications.

### 3.2. Production Yield of Microparticles

**Figure 3:**
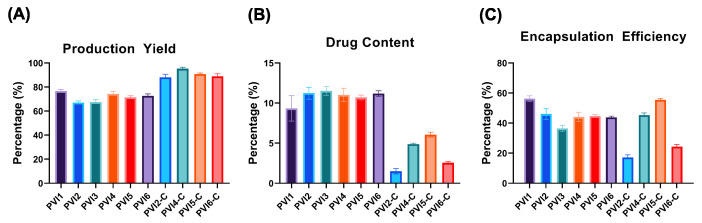
Graphical representation of: (**A**) Production Yield, (**B**) Drug Content, and (**C**) Encapsulation Efficiency of fabricated microparticles.

Figure 3A presents the production yield (PY) of the various fabricated microparticle formulations. All formulations achieved a yield above 65%, with PVI1 being the highest at approximately 77%, likely due to its higher PLGA content compared to the other formulations. PVI4 followed closely, which can be attributed to its high ICG concentration. The coated microparticles exhibited an even higher PY, primarily due to the absence of solvents that could cause the dissolution of the PLGA microparticles during the coating process.

The PY of the microparticles is influenced by the polymer-drug-ICG composition and the solvent evaporation rate. In this study, a consistent evaporation rate was maintained by standardizing the homogenization speed (rpm) and stirring conditions. At the current scale, the PY of the microparticles aligned with expectations; however, uncertainties may arise during upscaling. Larger batch volumes often require higher-capacity homogenizers, which may generate excess heat. This can accelerate solvent evaporation, potentially leading to inconsistent particle formation and thermal degradation of the core polymer. Additionally, increased temperatures may affect the stability of the encapsulated drug and ICG, reducing their efficacy. To address these challenges, process optimizations such as implementing external cooling systems, adjusting solvent composition, or using alternative homogenization techniques (e.g., high-pressure homogenization) could be explored. Future studies should focus on evaluating the impact of scaling parameters on microparticle integrity and drug release profiles to ensure reproducibility at larger scales.

### 3.3. Percent Drug Content and Encapsulation Efficiency

The drug content (DC) and encapsulation efficiency (EE) of the fabricated microparticles are shown in Figure 3B,C. PVI1 exhibited the lowest DC (9.35 ±1.6%) but the highest EE (56.2 ±1.95%), likely due to its smaller quantity of drugs compared to other formulations. In contrast, increasing the drug concentration in PVI3 did not improve the percent DC (11.54 ±0.6%) but reduced the EE (36.6 ±1.98%).

Therefore, PVI2, which had an intermediate drug concentration, was selected as the optimal formulation. Similar formulations based on PVI2 (PVI4, PVI5, and PVI6), which varied in ICG concentrations, displayed comparable DC and EE. A significant decrease in DC was observed in the coated particles, likely due to drug dissolution into the buffer solution during the chitosan coating process. Notably, PVI5-C demonstrated higher EE than the other coated particles, aligning with its superior antibacterial performance, where it more effectively eliminated bacteria.

### 3.4. Characterization of Fabricated Microparticles

**Figure 4:**
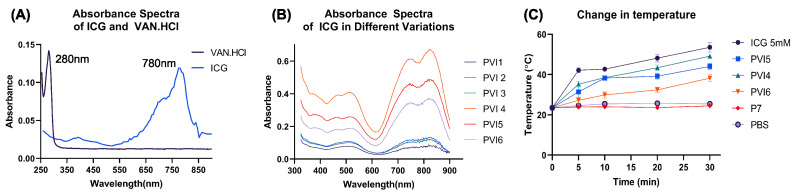
(**A**) Absorbance spectra of raw ICG and Van.HCl, (**B**) Absorbance spectra of Indocyanine Green in various PLGA-Van.HCl-ICG MP formulations, (**C**) Temperature change in various samples under NIR light exposure, showing significant photothermal activity of fabricated microparticles compared to control sample P7 and standalone PBS.

Figure 4A shows absorbance spectra of ICG in different formulations of PLGA-Van.HCl-ICG microparticles. The curves represent different microparticle compositions, affecting encapsulation efficiency, ICG concentration, and aggregation state.

Peaks in the near-infrared region (700–900 nm) are characteristic of ICG absorbance, with variations in intensity and spectral shifts reflecting differences in the formulation parameters of the microparticles. This confirms that ICG is successfully encapsulated in the PLGA microparticles, as the characteristic absorbance peaks of ICG are still visible. PVI4, PVI5, and PVI6 have the highest absorbance values, indicating a higher concentration of ICG in these formulations. The lower curves (PVI1, PVI2, and, PVI3) suggest lower ICG encapsulation affecting the optical properties.

Figure 4B shows the temperature change over time when the ICG-containing microparticles were exposed to NIR light, with other control samples for comparison. The temperature increases rapidly for the positive control (ICG 5 mM), PVI4, and PVI5 microparticles when exposed to NIR light, indicating significant photothermal activity. PVI6 also shows an increase in temperature but at a slower rate compared to the other formulations of microparticles. P7 shows similar results to the negative control (PBS), which confirms that there is no photo-thermal activity in the absence of ICG.

**Figure 5:**
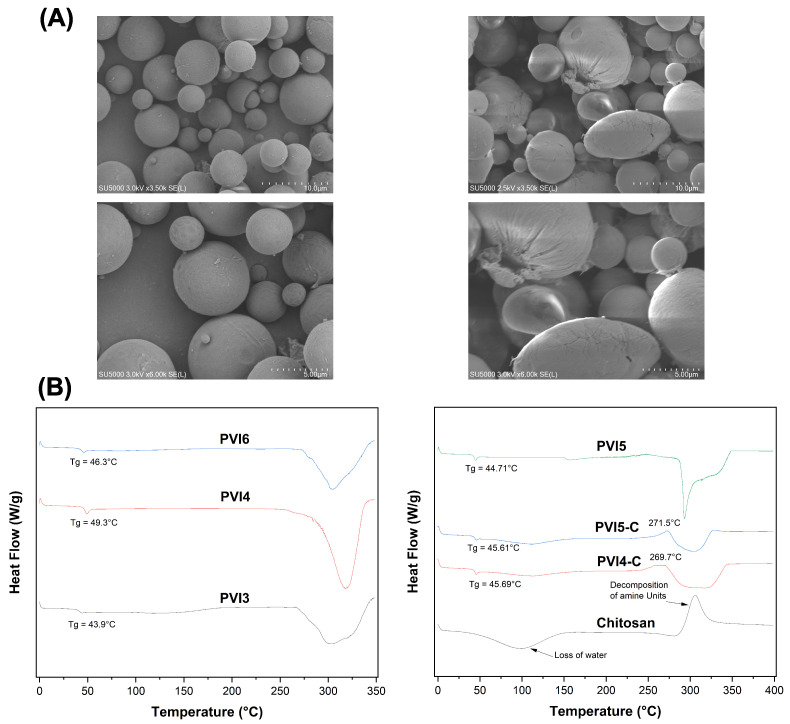
(**A**) SEM images uncoated (**Left**) and coated (**Right**) PLGA microparticles, (**B**) **Left**: DSC thermogram of uncoated microparticles (PVI3, PVI4, PVI6), **Right**: DScC thermogram of chitosan-coated microparticles, pure chitosan and uncoated PVI5.

Figure 5A presents SEM images of uncoated and coated PLGA microparticles.

The uncoated microparticles display a uniform size and shape with minimal surface irregularities, whereas the coated microparticles exhibit variable shapes and noticeable surface irregularities, indicating successful chitosan coating. The images reveal no significant size difference between the two types of microparticles, likely due to the short coating duration employed to minimize cargo diffusion. The heat flow curves for the different formulations are shown in Figure 5B.

The glass transition (Tg) temperatures for all formulations have been labeled below the curves. There is a negligible difference in the Tg for the different formulations, including the coated particles. The Tg is consistent because the core polymer is PLGA. From the graph, we can see that while the uncoated microparticles show negligible exothermic reaction around 270 °C, the chitosan-coated microparticles do show an exothermic peak, which could be attributed to the presence of chitosan. In comparison, pure chitosan has an exothermic peak at approximately 305 °C. The glass transition temperature (Tg) of PLGA microparticle formulations is a key factor influencing their drug release properties but is not as commonly studied as the Tg of raw PLGA polymers. During emulsion-based fabrication, solvent removal induces a transition to a glassy state, similar to cooling below Tg, though this process is influenced by solvent diffusion and polymer chain dynamics [65]. Factors such as solvent removal rate, water uptake during extraction, drying conditions, and applied shear stress during emulsion formation significantly affect the physical state of the microparticles [66]. Faster solvent removal traps higher energy, while slower removal allows structural relaxation, impacting encapsulation efficiency and stability. These process variables collectively influence microparticle morphology, porosity, and density, highlighting the importance of controlled fabrication conditions for achieving desired drug release profiles.

**Figure 6:**
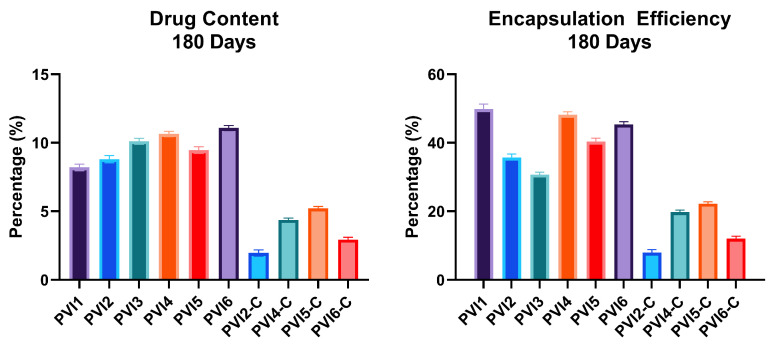
Graphical representation of: (**Left**) Drug Content, and (**Right**) Encapsulation Efficiency of fabricated microparticles 180 days after fabrication.

**Figure 7:**
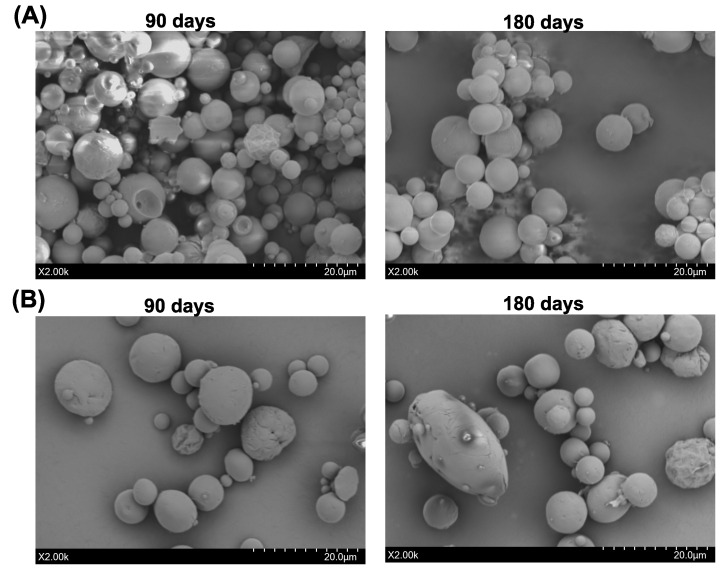
SEM images of (**A**) Uncoated and (**B**) Chitosan-coated PLGA microparticles 90 days (**Left**) and 180 days (**Right**) post-fabrication.

The results for DC and EE after 180 days of storage are presented in Figure 6. As shown, no significant differences were observed in DC and EE, indicating the stability of the drug delivery system over time. Additionally, Figure 7 displays SEM images taken at 90 and 180 days for both coated and uncoated microparticles. These images confirm that the structural integrity of the microparticles remained intact throughout the storage period, with no noticeable degradation or morphological changes.

These findings align with previous studies on PLGA-based drug delivery systems. For instance, Simon-Yarza et al. reported high encapsulation efficiencies for vascular endothelial growth factor encapsulated in PEG-PLGA microparticles, maintaining stability over extended periods [67]. Similarly, a study by Gasmi et al. demonstrated that encapsulating dexamethasone in PLGA microparticles resulted in sustained release profiles without significant changes in drug content over time [68]. Maintaining the structural integrity of microparticles is crucial for consistent drug release profiles. In our study, the SEM images at 90 and 180 days showed no significant morphological changes in both coated and uncoated microparticles. This observation is consistent with findings from a previous study, where it was observed that PLGA microparticles retained their spherical shape and surface morphology during storage, contributing to stable drug release kinetics [69]. Overall, the sustained DC and EE values, along with the preserved structural integrity observed in our study, underscore the potential of PLGA-based microparticles for long-term drug delivery applications.

### 3.5. FTIR Spectra of Chitosan-PLGA Particles to Confirm Chemical Conjugation of Chitosan to PLGA

**Figure 8:**
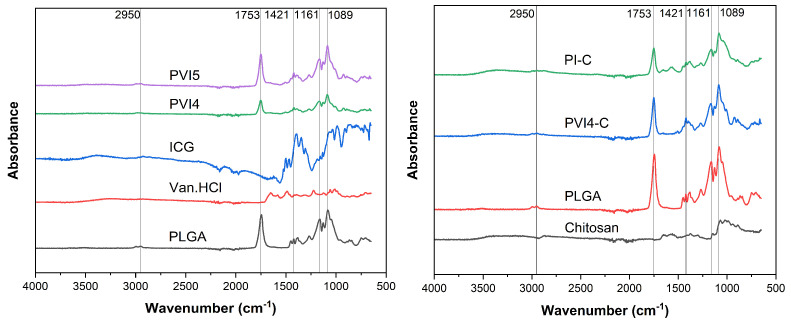
(**Left**) FTIR spectra of uncoated microparticles, bulk PLGA, free Van.HCl, and free ICG, (**Right**) FTIR spectra of chitosan-coated microparticles, pure chitosan and bulk PLGA.

FTIR results are shown in Figure 8. PLGA shows distinct FTIR peaks, including a strong peak around 1750 cm^−1^ corresponding to -COOH groups in PLGA. Other significant peaks include the region around 1180–1100 cm^−1^, associated with C–O stretching from the ester linkages [70,71].

These peaks clearly define the PLGA structure and are critical for identifying its presence in composite materials. The FTIR spectrum of free ICG reveals characteristic absorption bands. Aromatic C=O stretching vibrations are observed between 1400–1500 cm^−1^. Additionally, the benzene ring’s C-O-H bond shows two distinct peaks: one at 1150 cm^−1^, corresponding to bending vibrations, and another at 1390 cm^−1^, related to tensile vibrations [72]. Chitosan exhibits characteristic peaks in the FTIR spectrum, including a broad peak around 3420 cm^−1^ due to O–H and N–H stretching, a peak at 2925 cm^−1^ for C–H stretching, and notable peaks around 1650–1560 cm^−1^ corresponding to amide I and II bands (N–H bending and C=O stretching), and the vibration of -O-H and C-H in the ring is responsible for the absorption peaks at 1416 cm^−1^ and 1320 cm^−1^. Additionally, a prominent peak at 1080–1030 cm^−1^ arises from C–O–C stretching, typical of polysaccharides due to glycosidic linkages [73].

The presence of chitosan in the PVI4-C and PI-C samples is confirmed by the characteristic chitosan peaks, particularly the 1089 cm^−1^ peak (C–O–C stretching) and the shoulder near 1421 cm^−1^ (amide I and II vibrations). These appear alongside PLGA’s signature peak at 1753 cm^−1^ (C=O stretching), indicating that chitosan has been successfully conjugated to the PLGA particles. The combination of these peaks from both chitosan and PLGA in the composite spectra provides strong evidence of the coating or binding of chitosan to the PLGA particles. A summary of the FTIR spectra peaks is shown in Table 3.

### 3.6. Wettability Test

**Figure 9:**
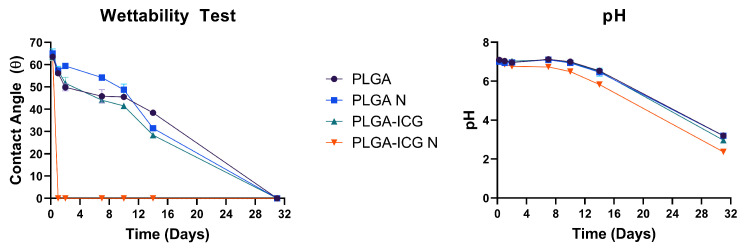
(**Left**) Graphical representation of the average contact angle, (**Right**) pH at different time points of the PBS solution that the films were submerged in. Note: PLGA N and PLGA-ICG N represent NIR irradiated samples.

The results for the contact angle and pH measurements are shown in Figure 9. There was a significant effect of subjecting NIR light to PLGA-ICG films, causing the film to break apart within 24 h of exposure. This is due to the heating of ICG, which led to maximum surface degradation with negligible bulk degradation of the film, resulting in a drop in an enormous drop in the contact angle. Furthermore, the molecular weight of the PLGA used for this study is low, subsequently leading to faster degradation. The pH readings do not have significant changes, which stipulates that there was not much PLGA subsequently causing the pH to drop. On day 31, the PBS in which the films were dispersed was highly acidic, which is because of the hydrolysis (degradation) of the polymer leading to the release of lactic and glycolic acids [74]. Due to the presence of a lower surface area in the PLGA films, the initial degradation is primarily restricted to the surface, whereas the microparticles have a higher surface area, which would facilitate bulk degradation and a quicker release of the drug that is present inside [75].

### 3.7. In Vitro Drug Release Assay

**Figure 10:**
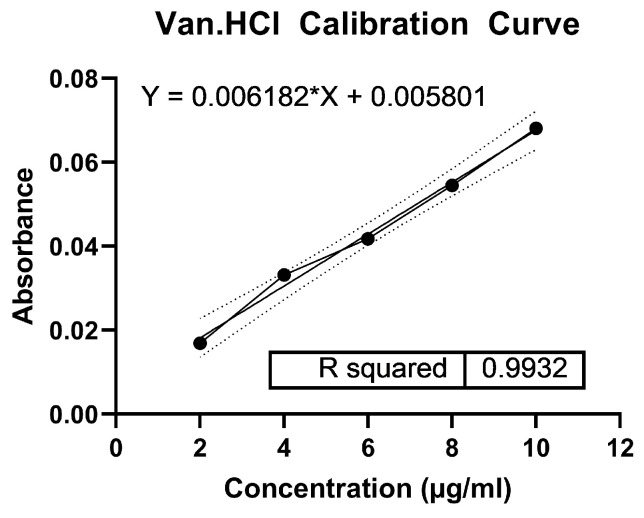
Calibration curve of vancomycin hydrochloride.

**Figure 11:**
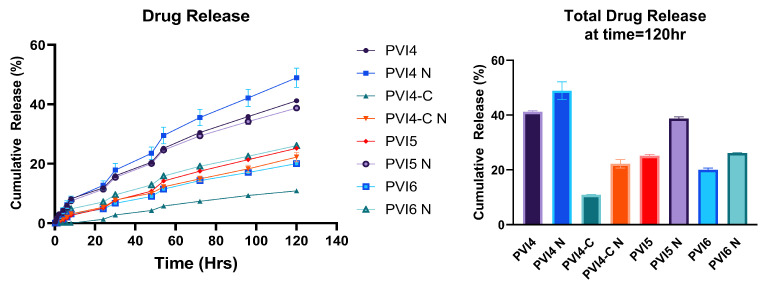
In vitro drug release assay results for different formulations of fabricated microparticles. (**Left**) Drug release results of different formulations from 0–120 h. (**Right**) A comparison of the total drug (at time T = 120 h) released between the coated and the uncoated microparticles.

The calibration curve for Van.HCl is shown in Figure 10. It was formulated by taking different concentrations of Van.HCl dissolved in PBS and taking the absorbance reading at 280 nm. The drug release results for the different formulations of fabricated particles (both coated and uncoated) are shown in Figure 11. A zero-order kinetic release was followed for all formulations, including the coated particles as shown in Table 4.

Zero-order kinetics pertains to a sustained and controlled release until the drug contained within the reservoir (microparticles) is depleted [76].

The irradiated PVI4 N exhibits the highest cumulative drug release, showing a significant and steady increase throughout the experiment. Non-irradiated PVI4 also shows high drug release, though slightly lower and reaching a plateau earlier than the PVI4 N, suggesting the effect NIR light has on making a difference in drug diffusion rates.

In comparison, PVI5 and PVI6 microparticles, which lack any stimulation of ICG, show moderate release, indicating that a higher concentration of ICG enhances drug delivery. Notably, the chitosan-coated microparticles demonstrated unsubstantial drug release, likely because the chitosan coating acts as a barrier and slows the release rate, which makes it suitable for sustained drug delivery. Chitosan further prevents the bulk degradation of the microparticles even when exposed to NIR light, slowing the release. This data suggests that ICG-incorporated microparticles, when exposed to NIR light, are effective in enhancing drug release, while chitosan-coated particles can provide a slower, controlled release.

Furthermore, the release kinetics of various PLGA microparticle formulations were evaluated using four models: *Zero-order*, *First-order*, *Second-order*, *Korsmeyer–Peppas (K–P)*, and *Hixson–Crowell (H–C)* [77,78]. Among these, the *Zero-order* model consistently showed the highest coefficient of determination ($R^{2}\approx 0.97$–0.99) across all formulations, both with and without NIR exposure, indicating a predominantly constant drug release rate over time. This suggests that the drug release is independent of concentration and points to a controlled release mechanism, possibly driven by matrix erosion or diffusion-limited release from the polymer matrix. In contrast, both *First-order* and *Second-order* models demonstrated poor fits to the data ($R^{2}$ ranging from 0.13 to 0.71), indicating that concentration-dependent release mechanisms were not dominant in these systems.

The *Korsmeyer–Peppas* model provided a good fit in selected formulations, especially PVI4 ($R^{2}=0.99$) and PVI5 ($R^{2}=0.78$), but was less predictive in NIR-exposed conditions, particularly for PVI4 N ($R^{2}=0.64$) and PVI5 N ($R^{2}=0.60$). This suggests that while diffusion-based mechanisms may contribute to the release under native conditions, the application of NIR light may alter the release kinetics, potentially by modifying the microparticle structure or enhancing polymer degradation. The release exponent *n* values for several groups (e.g., PVI4-C and PVI5) were greater than 0.89, indicating a *Super Case-II transport*, which implies that swelling, relaxation of polymer chains, and erosion collectively contribute to drug release [79].

The *Hixson–Crowell* model, which describes drug release from systems where there is a change in surface area and diameter over time, showed moderate $R^{2}$ values (0.69–0.86). This moderate fit suggests that surface erosion of PLGA microparticles plays a role in drug release but is not the dominant mechanism [80]. Interestingly, the values of $k_{\mathrm{kp}}$ (release rate constant for the K–P model) varied significantly across formulations, with values spanning from $6.9\times 10^{-11}$ to 2.16, indicating that formulation modifications and NIR exposure substantially influence the rate of release. For example, PVI4 (without NIR) had a much higher $k_{\mathrm{kp}}$ (2.16) compared to its NIR-exposed counterpart ($8.8\times 10^{-5}$), suggesting that NIR exposure may transiently inhibit diffusion or alter polymer–drug interactions in this case. Overall, these findings indicate that the *Zero-order* and *Korsmeyer–Peppas* models best describe the release behavior of the PLGA microparticles, with NIR exposure modulating release kinetics in a formulation-dependent manner. These observations support the potential of these microparticles for achieving sustained, tunable drug delivery, particularly when integrated with light-responsive components.

As noted before, the release of drugs from PLGA microparticles depends on the molecular weight, composition: Lactic Acid- Glycolic Acid ratio (LA-GA) [81,82], and Tg of the polymer [83,84]. For the coated particles, previous studies have reported that the chitosan coating effectively reduces the burst release effect of PLGA microparticles [85]. This observation is consistent with the drug release results obtained in this study. Chitosan coating plays a crucial role in modulating drug release, even under NIR irradiation, by forming polyelectrolyte complexes and exhibiting pH-responsive behavior. In this study, the cumulative release at 120 h for NIR-irradiated PVI4-C (chitosan-coated microparticles) was 22.26%, whereas, for uncoated PVI4 microparticles, it reached 41.23%, demonstrating the effectiveness of chitosan in preventing burst release, which is seen commonly in PLGA delivery systems [86]. Chitosan, a cationic polysaccharide, interacts with negatively charged biomolecules to form polyelectrolyte complexes, which enhance drug encapsulation and create a diffusion barrier, reducing premature drug leakage and ensuring sustained release [87,88]. Additionally, chitosan’s pH-sensitive nature further controls drug release, as it remains protonated and soluble in acidic environments but transitions into an insoluble, gel-like state at physiological pH, restricting drug diffusion [89,90]. The presence of ICG, a NIR-absorbing dye, enables photothermal activation upon exposure to NIR light (808 nm), generating localized heat that can soften the polymer matrix and facilitate drug diffusion [91]. However, despite this heat-induced release mechanism, the chitosan coating effectively reduces photothermal-triggered burst release, as it acts as a secondary barrier that stabilizes drug diffusion [92]. These findings confirm the efficiency of chitosan in controlled drug delivery applications and suggest that optimizing coating thickness, crosslinking density, and formulation strategies could further enhance its performance in sustained and targeted drug release systems. Future studies should focus on fine-tuning these parameters to improve long-term drug stability and release kinetics under NIR-triggered conditions.

### 3.8. Antibacterial Tests

**Figure 12:**
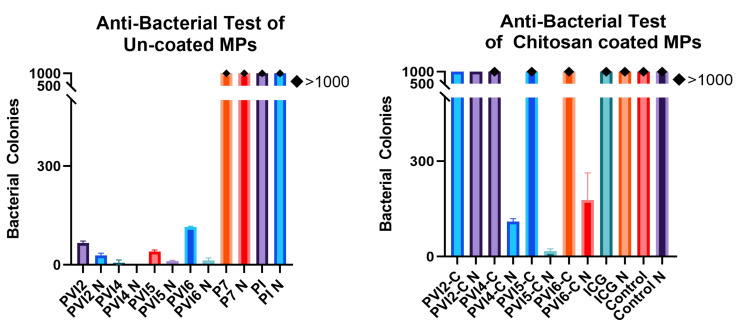
Antibacterial Tests: (**Left**) Results from tests with uncoated microparticles. (**Right**) Antibacterial test results from chitosan-coated microparticles. Note: Results with exposure to NIR light have been labeled with “N” at the end such as PVI4 N, Control N, ICG N, PVI4-C N, etc.

The results for the antibacterial tests on both coated and uncoated microparticles are shown in Figure 12. The antibacterial activity of the microparticles was influenced by both drug content and ICG content in conjunction with exposure to NIR light. Samples exposed to NIR light consistently demonstrated enhanced drug release compared to their unexposed counterparts. The sample PVI4 exhibited the highest antibacterial activity, with an average of 11 colonies observed in the unexposed group and no colonies in the NIR-exposed group. Significant bacterial growth was observed in the P7 and PI groups.

The chitosan-coated microparticles effectively slowed drug release, thereby reducing antibacterial activity. However, the NIR-exposed coated microparticles displayed a marked improvement in antibacterial activity compared to the unexposed samples, as indicated by significantly less bacterial growth. When exposed to NIR light, PVI4-C N and PVI5-C N microparticles demonstrated significant resistance to bacterial growth, whereas their non-irradiated counterparts exhibited over 1000 bacterial colonies. Similarly, chitosan-coated PVI2 microparticles could not produce the same results as their counterparts, with more than 1000 bacterial colonies detected. This is primarily due to the amount of ICG present and its role in stimulated release when exposed to NIR light. The concentration of ICG in PVI2 is 500 μm, which is five times and ten times less than PVI5 and PVI4, respectively. To prevent substantial loss of Van.HCl and ICG into the solution while the microparticles were being coated with chitosan, the coating process was run for 8 h. Chitosan is said to have anti-bacterial properties [93], and chitosan-based nanoparticles [94] and micromotors [95] have been used for anti-bacterial experiments. Therefore, chitosan-coated microparticles are expected to exhibit inherent antibacterial properties. However, the coating process, lasting only 8 h, resulted in a thin layer of chitosan on the surface, which is insufficient to effectively eliminate bacteria. Low molecular weight (LMW) chitosan was utilized for the coating, and the combination of its LMW properties with the minimal chitosan concentration is consistent with previous research findings on bacterial growth [96,97].

### 3.9. Biocompatibilty Studies of Microparticles

**Figure 13:**
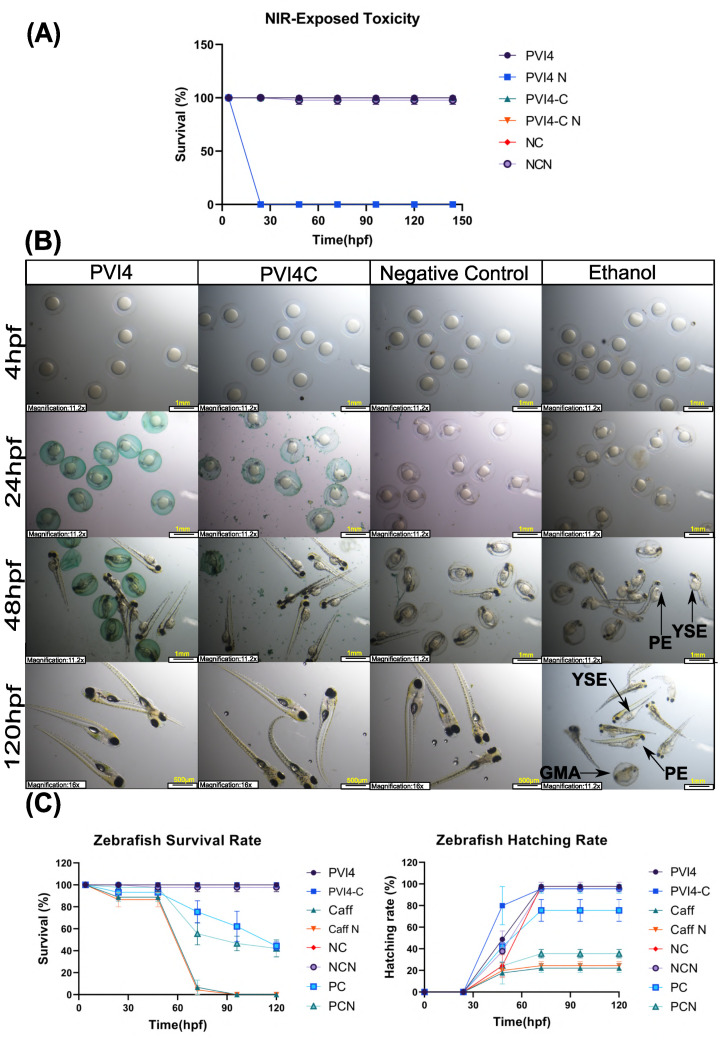
(**A**) ICG cytotoxicity assay for cytocompatibility, (**B**) microscopic images of zebrafish embryos (0–120 hpf) treated with fabricated microparticles (PVI4, PVI4-C), negative control and 2% (*v*/*v*) ethanol. Growth abnormalities such as yolk sac edema (YSE), pericardial edema (PE), and gross morphological abnormalities are indicated by black arrows, (**C**) percentage survival (**Left**) and hatching (**Right**) rates of zebrafish embryos over time (0–120 hpf) (mean + SD; n = 3).

The biocompatibility results are presented below in Figure 13. The microparticle variation containing the highest concentration of ICG was selected for biocompatibility testing using a Zebrafish Embryo Toxicity (ZET) model. The results indicate that the survival rate of zebrafish embryos (ZE) exposed to the microparticles was comparable to that of the negative control group, suggesting minimal toxicity of the microparticles. Interestingly, the hatching rate of the embryos exposed to the microparticles was faster compared to those in the negative control group, potentially indicating a positive interaction between the microparticles and the biological system.

Furthermore, no signs of gross malformations such as yolk sac edema, pericardial edema, or general morphological abnormalities were observed in the embryos exposed to the microparticles, implying that the microparticles do not adversely affect embryonic development. In contrast, zebrafish embryos treated with ethanol (used as a positive control) exhibited clear signs of developmental toxicity, including visible GMA, YSE, and PE, as highlighted in Figure 13. These findings suggest that the microparticles, even at the highest ICG concentration, demonstrate good biocompatibility with minimal toxic effects on zebrafish development.

However, in a separate trial, when the microparticles containing ICG were exposed at higher concentrations or for prolonged periods, they induced embryo death within the exposure window, signaling potential toxicity. This observed cytotoxicity could be harnessed as a beneficial feature in cancer treatment. The selective toxicity towards rapidly dividing cells, such as embryonic or cancer cells, suggests that these ICG-loaded microparticles could be promising candidates for targeted cancer therapy. Their ability to kill cells upon prolonged exposure hints at a potential mechanism for inducing cancer cell death while minimizing harm to surrounding healthy tissues when used in a controlled, localized manner.

**Figure 14:**
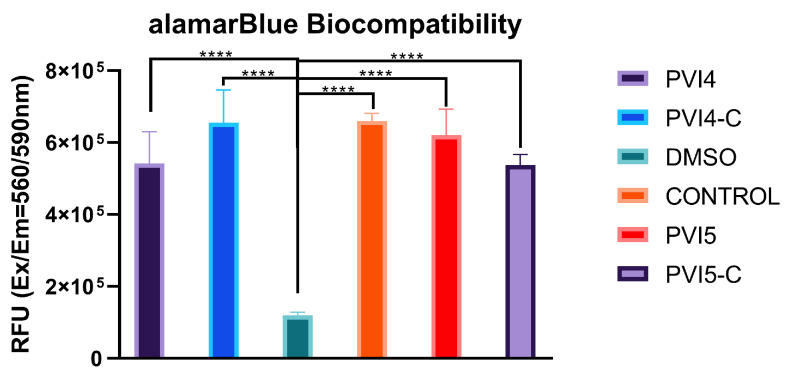
Cell viability after 24-h exposure to PVI4, PVI4-C, PVI5, and PVI5-C microparticles, with and without NIR irradiation (0.3 W/cm^2^, 808 nm). Viability was assessed using the alamarBlue™ assay, with absorbance measured at 560 nm/590 nm. Positive control: 3% DMSO; negative control: untreated cells. Note: **** indicates statistically significant difference with *p* < 0.0001.

Significant differences in cell viability were observed between the experimental groups (PVI4, PVI4-C, PVI5, PVI5-C) and the positive control (3% DMSO), with all groups showing significantly higher viability (*p* < 0.0001) when assessed by one-way ANOVA as shown in Figure 14. This indicates the biocompatibility of the microparticles, as their treatment did not induce cytotoxicity at the tested concentrations. The absence of any severe cytotoxic effects, even after prolonged exposure, further supports the biocompatibility of these particles. This is crucial for potential in vivo applications where the interaction of materials with biological systems must not compromise cell health or function. The modified versions (PVI4-C and PVI5-C) also maintained this biocompatibility, reinforcing the idea that surface modifications, such as chitosan coating, did not negatively impact the safety of the microparticles.

These findings demonstrate that the formulations are non-toxic under the conditions tested, thus making them promising for drug delivery or other therapeutic applications that require prolonged interaction with cells. Among the microparticles, the NIR-irradiated groups (PVI4 N, PVI4-C N, PVI5 N, and PVI5-C N) along with the positive control demonstrated the most pronounced reduction in cell viability compared to its non-irradiated counterpart and the untreated cells. Furthermore, NIR irradiation resulted in a decrease in cell viability for all microparticles (PVI4, PVI4-C, PVI5, and PVI5-C), with PVI5-C showing the greatest effect.

## 4. Conclusions and Future Direction

This study highlights the innovative potential of using PLGA microparticles combined with NIR-sensitive materials for targeted drug delivery. By integrating Van.HCl and ICG into PLGA microparticles and modifying their surfaces with chitosan, we achieved light-responsive control over drug release. Dynamic Light Scattering (DLS) analysis revealed that uncoated microparticles had sizes ranging from 2.26 to 6.78 μm, with low to moderate polydispersity indices (PI: 0.196–0.380), indicating good size uniformity. Upon chitosan coating, particle sizes increased substantially (up to 7.75 μm for PVI4-C), and PI also rose (up to 0.755), consistent with the formation of a hydrated polymer shell and interparticle interactions. ZP measurements confirmed strong negative surface charges for uncoated particles (as low as –70.7 mV for PVI1), shifting to positive values after chitosan coating (up to +41.01 mV for PVI6-C), confirming successful surface modification. These physicochemical changes are expected to influence drug release kinetics and biological interactions. Indeed, the chitosan coating significantly suppressed premature drug leakage, enabling controlled release only upon NIR exposure. Additionally, drug release studies fitted to kinetic models such as Korsmeyer–Peppas and Higuchi revealed diffusion-based release behavior and further supported the role of NIR irradiation in enhancing drug release rates. The addition of a chitosan coating further refined the release control, preventing drug release in the absence of light exposure. Biocompatibility testing using zebrafish embryos up to 120 hpf showed no significant morphological defects or developmental abnormalities, confirming the safety of the microparticle formulations for early-stage biological applications. These findings underscore the feasibility and promise of smart, light-responsive drug delivery systems, paving the way for more effective and personalized treatments. In general, the goal of creating a drug delivery system that can restrict the release of its contents for a given amount of time was achieved. Although the chitosan coating was able to reduce the rate of release, the shortcoming lies in the release of drugs without stimulation. Although minimal, this release could present a significant challenge for highly toxic drugs, underscoring the necessity of developing precise and controlled delivery mechanisms. In future studies, different coatings will be studied to contain the release, and a higher molecular weight PLGA polymer will be used to limit the porosity of the carriers. The drug release profiles of PLGA microparticles were best described by the zero-order and Korsmeyer–Peppas models, indicating controlled and diffusion-influenced release mechanisms. NIR exposure altered release kinetics in a formulation-dependent manner, demonstrating the potential for externally modulated, sustained drug delivery. These findings highlight the versatility of the fabricated microparticles for applications requiring precise control over therapeutic release.

The antibiotic Van.HCl can be substituted with potent anti-cancer drugs for release on the tumor site. While systematic release was achieved, the targeting aspect needs to be resolved. This can be done with further surface modifications, subsequently targeting the niche of cancer cells. We believe that the fabricated system will be a good foundation to be able to create a drug delivery system with controlled and targeted release upon exposure to NIR light.

## Figures and Tables

**Table 1:** Varying concentrations of initial polymer/drug/ICG for fabrication of different formulations. PLGA, and Van.HCl content in mg; ICG content in molarity; DCM and PVA solution content in mL.

	PVI1	PVI2	PVI3	PVI4	PVI5	PVI6	P7	PI
PLGA	250	225	200	225	225	225	300	300
Van.HCl	50	75	100	75	75	75	0	0
ICG (Molarity)	500 μM	500 μM	500 μM	5 mM	2.5 mM	1 mM	0	5.5 mM
PVA Solution	60	60	60	60	60	60	60	60
DCM	9	9	9	9	9	9	10	10

**Table 2:** Zeta Potential, Z-average diameter and Polydispersity Index (PI) values obtained from DLS analysis of various formulations of fabricated microparticles. Measurements were performed at a backscatter angle of 173° using a Malvern Zetasizer Advance Ultra.

	Zeta Potential (mV)	Z-Average (μm)	PI
PVI1	−70.70 ±1.42	2.26	0.284
PVI2	−55.23 ±1.01	2.44	0.380
PVI2-C	34.83 ±0.55	5.71	0.454
PVI3	−42.80 ±1.15	5.21	0.264
PVI4	−56.50 ±0.44	2.34	0.196
PVI4-C	14.47 ±1.38	7.75	0.755
PVI5	−59.27 ±1.81	6.78	0.324
PVI5-C	27.17 ±0.72	7.70	0.686
PVI6	−44.67 ±3.86	3.34	0.293

**Table 3:** FTIR analysis of ingredients used in the formulation of microspheres PLGA, Van.HCl, ICG, and different formulations: PVI4, PVI4-C.

Peak Observed (cm^−1^)	Functional Group
**FTIR of PLGA**	
3400–3500	OH end group
1750	–COOH groups
1100–1800	C–O stretching
**FTIR of Van.HCl**	
3400	stretching of phenolic OH
1650	aromatic C=C
1505	C=O secondary amide function
1390	C–O phenolic O–H group
**FTIR of ICG**	
1400–1500	C=O stretches
1150 and 1390	C–O–H bond on benzene ring
**FTIR of PVI4**	
1750	–COOH groups
1400–1500	C=O stretches
1100–1800	C–O stretching
**FTIR of Chitosan** 3420	OH and NH stretching
2925	C–H stretching
1650–1560	Amide I and II
**FTIR of PVI4-C**	
1750	–COOH groups
1089	C–O–C stretching
1421	Amide I and II

**Table 4:** Model application to the release profile data of different formulations of PLGA microparticles.

Kinetic Model	PVI4	PVI4 N	PVI4-C	PVI4-C N	PVI5	PVI5 N	PVI6	PVI6 N
***Zero***								
R^2^	0.97	0.99	0.99	0.99	0.99	0.97	0.99	0.98
***1st***								
R^2^	0.71	0.24	0.67	0.27	0.36	0.2	0.36	0.27
***2nd***								
R^2^	0.28	0.13	0.63	0.20	0.29	0.13	0.3	0.20
**K-P** *								
R^2^	0.99	0.64	0.78	0.71	0.78	0.6	0.78	0.7
$k_{kp}$	2.16	8.8 $\times \;10^{-5}$	6.9 $\times \;10^{-11}$	1.3 $\times \;10^{-6}$	4.9 $\times \;10^{-8}$	9.1 $\times \;10^{-5}$	5 $\times \;10^{-8}$	1.5 $\times \;10^{-6}$
N	0.61	3.5	5.5	4.4	5.1	3.5	5	4.4
**H-C** **								
R^2^	0.84	0.79	0.86	0.71	0.77	0.69	0.74	0.7
$k_{x}$	0.02	0.026	0.02	0.02	0.02	0.02	0.02	0.02

* K-P: Korsmeyer–Peppas model ($k_{kp}$: release rate constant); ** H-C: Hixson–Crowell model (k*^x^*: rate constant).

## Data Availability

The datasets generated during and/or analysed during the current study are available from the first and corresponding author on reasonable request.

## Abbreviations

The following abbreviations are used in this manuscript:

PLGA	Poly(lactic-co-glycolic acid)
Van.HCl	Vancomycin Hydrochloride
ICG	Indocyanine Green
NIR	Near Infrared
DCM	Dichloromethane
FBS	Fetal Bovine Serum
SEM	Scanning Electron Microscopy
DLS	Dynamic Light Scattering
ZP	Zeta Potential
DSC	Differential Scanning Calorimetry
FTIR	Fourier Transform Infrared
